# Supplementary material for: Thermal Conductivity of Metastable Ionic Liquid [C2mim][CH3SO3]
Source: Molecules. 2020 Sep 18;25(18):4290. doi: 10.3390/molecules25184290 (PMC7570973; doi:10.3390/molecules25184290)
Supplement: Supplementary file 1 [file molecules-25-04290-s001.pdf]

Manuscript ID: molecules-896276

Thermal Conductivity of Metastable Ionic Liquid $[C_2mim][CH_3SO_3]$

D. Lozano-Martín, S.I.C. Vieira, X. Paredes, M.J.V. Lourenço, C.A. Nieto de Castro, J. V. Sengers and K. Massonne

Table 1. – Thermal conductivity of $[C_2mim][CH_3SO_3]$ as a function of temperature. For the liquid and metastable liquid, the water free values are also displayed.

Date	Exp. #	λ /W·m^{-1}·K^{-1}	ΔT_{rise}/ K	T_{ref} / K	λ_{wf} /W·m^{-1}·K^{-1}	Date	Exp. #	λ /W·m^{-1}·K^{-1}	ΔT_{rise}/ K	T_{ref} / K	λ_{wf} /W·m^{-1}·K^{-1}
	Sample 1						Sample 2				
31/10/2019	1	0.2939	0.82	273.97		21/11/2019	110	0.2318	0.17	273.32	
	2	0.2940	0.82	273.97			111	0.2430	0.94	274.09	
	3	0.2947	0.75	273.90			113	0.2407	0.99	274.14	
	4	0.2955	0.82	273.97			114	0.2547	0.89	274.04	
	5	0.2952	0.80	273.95		22/11/2019	115	0.2503	0.79	273.94	
	6	0.2981	0.82	273.97			116	0.2504	0.78	273.93	
	7	0.2877	0.70	273.85			117	0.2615	0.69	273.84	
	8	0.2907	0.80	273.95			118	0.2707	0.86	274.01	
	9	0.2911	0.77	273.92			119	0.2865	0.79	273.94	
	10	0.2932	0.69	273.84			120	0.2694	0.69	273.84	
	11	0.2937	0.75	273.90			127	0.3057	0.80	273.95	
04/11/2019	1	0.2852	0.69	273.84			128	0.3055	0.82	273.97	
	2	0.2857	0.69	273.84			129	0.3094	0.72	273.87	
	3	0.2862	0.79	273.94			130	0.3141	0.62	273.77	
	4	0.2864	0.72	273.87			131	0.3048	0.80	273.95	
	5	0.2892	0.70	273.85		25/11/2019	132	0.3284	0.72	273.87	
	6	0.2882	0.69	273.84			133	0.3309	0.72	273.87	

	7	0.2888	0.67	273.82
	8	0.2938	0.75	273.90
	9	0.2965	0.67	273.82
	10	0.2947	0.69	273.84
	11	0.2979	0.65	273.80
05/11/2019	12	0.2891	0.77	273.92
	13	0.2954	0.67	273.82
	14	0.2967	0.72	273.87
	15	0.3087	0.57	273.72
	16	0.3035	0.64	273.79
	17	0.3029	0.60	273.75
	18	0.2979	0.69	273.84
	19	0.3001	0.67	273.82
	20	0.3011	0.67	273.82
	21	0.3031	0.64	273.79
06/11/2019	22	0.3032	0.59	273.74
	23	0.3025	0.65	273.80
	24	0.3025	0.62	273.77
	25	0.3050	0.62	273.77
	26	0.3045	0.59	273.74
	27	0.3131	0.55	273.70
	28	0.3137	0.60	273.75
	29	0.3175	0.51	273.66
	30	0.3194	0.54	273.69
	31	0.3242	0.52	273.67
07/11/2019	32	0.3169	0.62	273.77
	33	0.3217	0.60	273.75
	34	0.3238	0.55	273.70
	35	0.3240	0.54	273.69
	134	0.3416	0.60	273.75
	135	0.3314	0.72	273.87
	136	0.3397	0.62	273.77
	137	0.3501	0.60	273.75
	138	0.3571	0.60	273.75
	139	0.3593	0.60	273.75
	140	0.3581	0.64	273.79
	141	0.3685	0.54	273.69
26/11/2019	142	0.3616	0.68	273.83
	143	0.3702	0.57	273.72
	144	0.3800	0.55	273.70
	145	0.3717	0.63	273.78
	146	0.3720	0.63	273.78
	147	0.3791	0.62	273.77
	148	0.3861	0.59	273.74
	149	0.3901	0.59	273.74
	150	0.3975	0.47	273.62
	151	0.3952	0.57	273.72
27/11/2019	152	0.3867	0.59	273.74
	153	0.3922	0.57	273.72
	154	0.3910	0.55	273.70
	155	0.3986	0.49	273.64
	156	0.4025	0.49	273.64
	157	0.3881	0.57	273.72
	158	0.4041	0.54	273.69
	159	0.4065	0.54	273.69
	160	0.4060	0.55	273.70
	161	0.4037	0.54	273.69
28/11/2019	162	0.3518	0.57	273.72

	36	0.3295	0.52	273.67			163	0.3514	0.55	273.70	
	37	0.3117	0.63	273.78			164	0.3507	0.58	273.73	
	38	0.3200	0.54	273.69			165	0.3529	0.55	273.70	
	39	0.3174	0.55	273.70			166	0.3487	0.58	273.73	
	40	0.3196	0.54	273.69			167	0.3237	0.52	273.67	
	41	0.3113	0.62	273.77			168	0.3181	0.54	273.69	
08/11/2019	42	0.2069	0.78	273.93	0.2038		169	0.2950	0.68	273.83	
	43	0.2287	0.21	273.36	0.2256		170	0.2872	0.42	273.57	
	44	0.2067	0.73	273.88	0.2036		171	0.2667	0.71	273.86	
	45	0.2199	0.32	273.47	0.2168	29/11/2019	172	0.1977	1.17	274.32	0.1946
	46	0.2207	0.42	273.57	0.2176		173	0.1966	1.25	274.40	0.1935
	47	0.2021	0.91	274.06	0.1990		174	0.1963	1.25	274.40	0.1932
	48	0.2005	1.02	274.17	0.1974		175	0.1964	1.25	274.40	0.1933
	49	0.1996	0.84	273.99	0.1965		176	0.1974	1.23	274.38	0.1943
	50	0.2034	1.13	274.28	0.2003		177	0.1935	1.27	274.42	0.1904
	51	0.2233	0.32	273.47	0.2202		178	0.1964	1.22	274.37	0.1933
11/11/2019	52	0.1983	1.15	274.30	0.1953		179	0.1979	1.23	274.38	0.1948
	53	0.1995	1.13	274.28	0.1964		180	0.1963	1.25	274.40	0.1932
	54	0.2010	1.08	274.23	0.1979		181	0.1960	1.23	274.38	0.1929
	55	0.1984	1.23	274.38	0.1953	02/12/2019	182	0.1965	1.25	274.40	0.1934
	56	0.1989	1.17	274.32	0.1958		183	0.1977	1.23	274.38	0.1946
	57	0.1932	1.26	274.41	0.1901		184	0.1983	1.18	274.33	0.1952
	58	0.2010	1.21	274.36	0.1979		185	0.1975	1.23	274.38	0.1944
	59	0.2001	1.13	274.28	0.1970		186	0.1974	1.25	274.40	0.1943
	60	0.1984	1.18	274.33	0.1953		187	0.1971	1.20	274.35	0.1940
	61	0.1986	1.23	274.38	0.1955		188	0.1965	1.20	274.35	0.1935
12/11/2019	62	0.1964	1.22	274.37	0.1933		189	0.1950	1.25	274.40	0.1919
	63	0.1988	1.22	274.37	0.1957		190	0.1970	1.18	274.33	0.1939
	64	0.1993	1.22	274.37	0.1962		191	0.1976	1.18	274.33	0.1945

	65	0.1984	1.24	274.39	0.1953	03/12/2019	192	0.1931	1.13	274.28	0.1900
	66	0.1966	1.24	274.39	0.1935		193	0.1984	1.16	274.31	0.1953
	67	0.1950	1.19	274.34	0.1919		194	0.1980	1.23	274.38	0.1949
	68	0.1977	0.91	274.06	0.1946		195	0.1987	1.16	274.31	0.1956
	69	0.1922	1.01	274.16	0.1891		196	0.1970	1.23	274.38	0.1939
	70	0.1986	0.87	274.02	0.1955		197	0.1972	1.16	274.31	0.1941
	71	0.1965	0.91	274.06	0.1935		198	0.1954	1.26	274.41	0.1923
	73	0.1980	0.90	274.05	0.1949		199	0.1977	1.11	274.26	0.1946
	74	0.1973	0.85	274.00	0.1942		200	0.1968	1.24	274.39	0.1937
13/11/2019	75	0.1883	0.77	273.92	0.1852		201	0.1961	1.21	274.36	0.1930
	76	0.1968	1.02	274.17	0.1937						
	77	0.1966	1.09	274.24	0.1935						
	78	0.1987	0.90	274.05	0.1956						
	79	0.2093	0.81	273.96	0.2062						
	80	0.2058	0.89	274.04	0.2027						
	81	0.2031	0.94	274.09	0.2000						
	82	0.2039	0.85	274.00	0.2008						
	83	0.2073	0.83	273.98	0.2043						
14/11/2019	85	0.1995	1.13	274.28	0.1964						
	86	0.1906	1.28	274.43	0.1875						
	87	0.1964	1.11	274.26	0.1933						
	88	0.1959	0.83	273.98	0.1928						
	89	0.1930	1.26	274.41	0.1899						
	90	0.1957	1.03	274.18	0.1926						
	91	0.2162	0.46	273.61	0.2131						
15/11/2019	92	0.2009	1.16	274.31	0.1978						
	93	0.2112	0.25	273.40	0.2081						
	94	0.1924	1.22	274.37	0.1893						
	96	0.1981	2.33	275.48	0.1950						

	97	0.1974	2.88	276.03	0.1943
	98	0.1981	1.90	275.05	0.1951
18/11/2019	99	0.1989	2.43	275.58	0.1958
	100	0.2048	0.94	274.09	0.2017
	101	0.2004	1.12	274.27	0.1973
	102	0.1968	1.15	274.30	0.1938
	103	0.1882	1.15	274.30	0.1851
	104	0.2016	1.05	274.20	0.1985
19/11/2019	105	0.1837	1.34	274.49	0.1806
	106	0.1856	1.09	274.24	0.1825
	107	0.1957	1.20	274.35	0.1926
	108	0.1974	1.04	274.19	0.1943
20/11/2019	109	0.1994	1.15	274.30	0.1963

[a] Expanded relative uncertainty U_r (λ) = 2 %, at a 95 % confidence level (k=2); U_r = 0.02 K
